# Supplementary material for: Antibacterial and Anti-Inflammatory Effects of Apolipoprotein E
Source: Biomedicines. 2022 Jun 17;10(6):1430. doi: 10.3390/biomedicines10061430 (PMC9220183; doi:10.3390/biomedicines10061430)
Supplement: Supplementary file 1 [file biomedicines-10-01430-s001.zip › Supplementary data and movies/Supplementary data.pdf]

Supplementary data

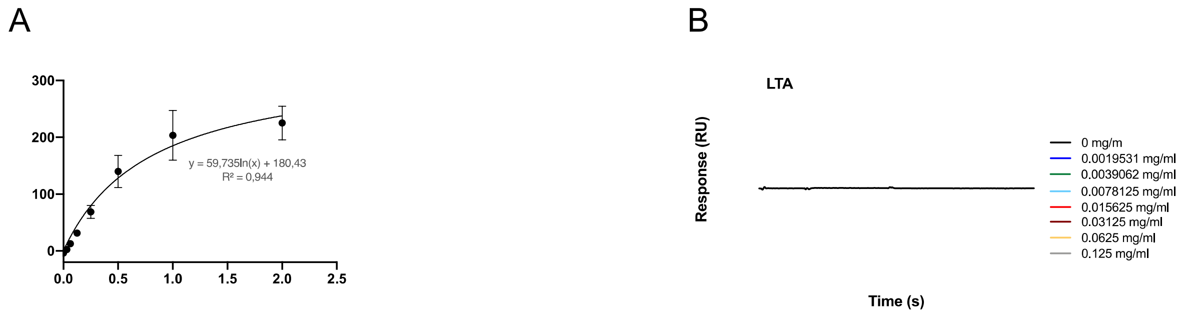

Figure S1. Biacore binding curves and negative control. (**A**) Biacore binding analysis between target analytes and immobilized APOE protein. Calibration curve that shows the response unit (RU) vs. concentration of LPS or lipid A. (**B**) Negative control LTA without biding affinity to immobilized APOE.

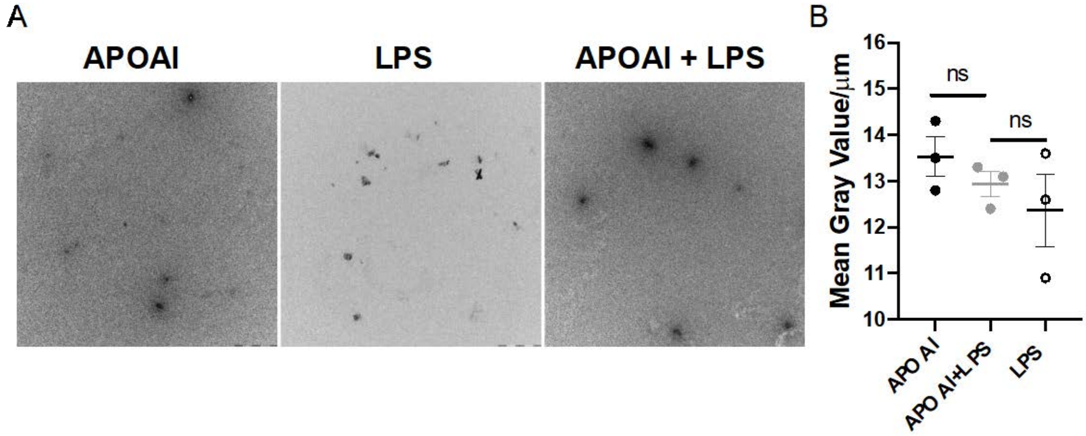

Figure S2. Visualization of macromolecular complexes of APOAI and LPS. (**A**) APOAI (2 μM) was incubated with 100 μg/ml LPS from *E. coli* for 30 min at 37°C. At the end of incubation, the sample composition was visualized by TEM. One representative image for each condition from three independent experiments is shown (n=3). Scale bar represents 1 μm. (**B**) Analysis of the complexes of APOAI and ligands following TEM. Quantification was performed using ImageJ 1.52k after all the images were converted to 8-bit, and the threshold was adjusted. The complexes of APOAI or LPS are expressed as the mean of gray value/μm ± SEM. In the graph, each point represents average of the measurements of one experiment. The graph represents the data from three independent experiments, after analysis of at least ten pictures per each experiment (n=3). Statistical analysis was performed using a one-way ANOVA with Dunnett's multiple comparison tests (ns = not significant).

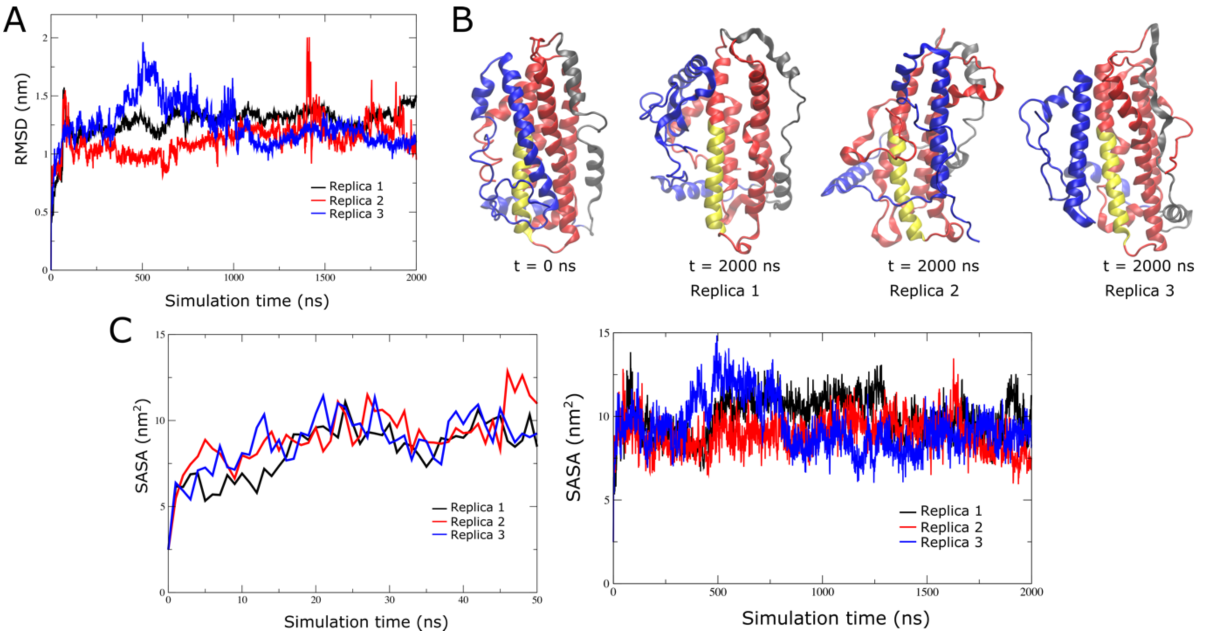

Figure S3. Molecular dynamics simulations of full-length APOE in explicit solvent. (**A**) Root mean square deviation (RMSD) of C-terminal domain backbone atoms over the simulation time across three replicas. Prior to the fit, a least-squares-fit was performed on all protein backbone atoms. (**B**) Initial and final snapshots of full length APOE in explicit solvent. These show the dissociation of the C-terminal domain to expose LDLR receptor binding region on helix 4 (residues 130-150). **C)** Solvent accessible surface area (SASA) of LDLR receptor binding region (residues 130-150) over the first 50 ns and over the entire 2,000 ns across three replicas. In (B) N-terminal domain is coloured in red with receptor binding region on helix 4 coloured in yellow, and C-terminal domain is coloured in blue with hinge region between domains shown in grey. Selected protein side chains and ligand molecules are shown in CPK sticks representation (cyan – carbon; red – oxygen; blue – nitrogen; brown – phosphorus; sulphur – yellow).

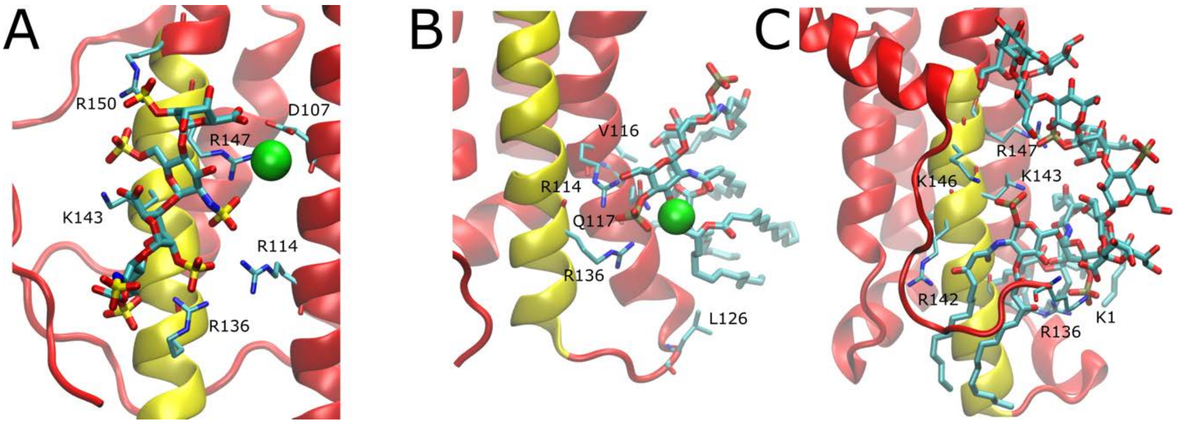

Figure S4. Molecular dynamics simulations of a docked ligand to a truncated APOE for: (**A**) heparin, (**B**) lipid A and **(C)** LPS. The final simulation snapshots are shown for systems initially derived from docking of a single molecule to the receptor binding region. N-terminal domain is coloured in red with receptor binding region on helix 4 coloured in yellow. Selected protein side chains and ligand molecules are shown in CPK sticks representation (cyan – carbon; red – oxygen; blue – nitrogen; brown – phosphorus; sulphur – yellow), with Mg^{2+} counterions shown as green spheres.

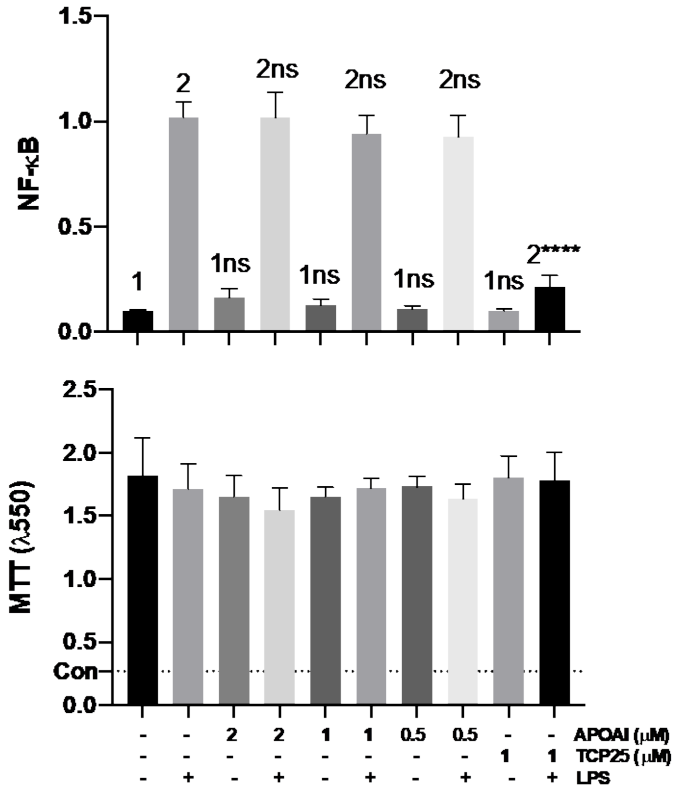

Figure S5. Anti-endotoxic effect of APOAI in vitro. THP-1-XBlue-CD14 cells were treated with APOAI (0.5, 1 and 2 μM), LPS (10 ng/ml) from *E. coli*, or a combination of both. 1 μM TCP25 was used as a positive control of NF- κB activation. MTT viability assay for analysis of toxic effects of APOAI on THP-1 cells. The dotted line (con) represents positive control of dead cells. The mean values of five measurements ± their SEM are shown. *P* values were determined using one-way ANOVA with Dunnett's multiple comparison test, **** = P ≤ 0.0001.

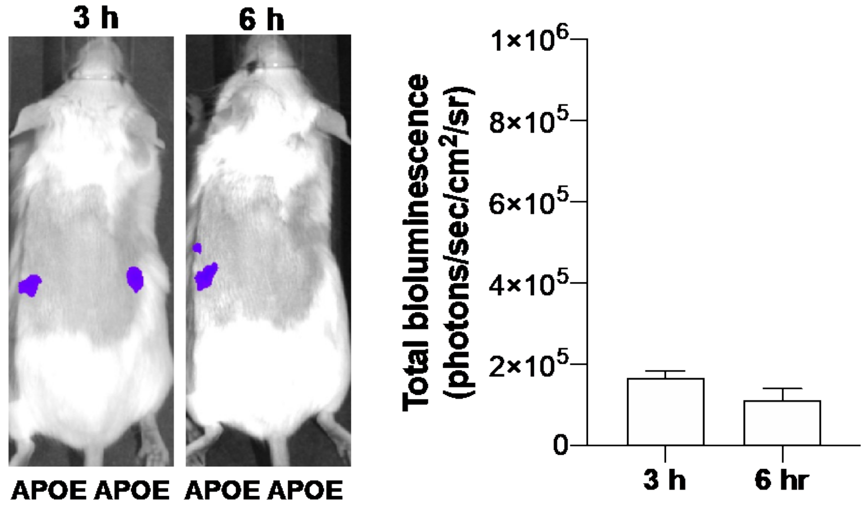

Figure S6. Immunomodulatory effect of APOE in vivo.

NF-κB activation in the NF-κB-RE-luc random transgenic mouse model was analyzed by the IVIS imaging. APOE (2 μM) (*left* and *right* side of the dorsum) was injected subcutaneously and the NF-κB response was longitudinally imaged 3 and 6 hours after the subcutaneous injection.

Movie S1. MD simulations of five lipid A molecules binding truncated APOE. N-terminal domain is

coloured in red with receptor binding region on helix 4 coloured in yellow. Protein side chains and lipid A molecules are shown in CPK sticks representation (cyan – carbon; red – oxygen; blue – nitrogen; brown – phosphorus; sulphur – yellow).

Movie S2. MD simulations of five LPS molecules binding truncated APOE. N-terminal domain is coloured in red with receptor binding region on helix 4 coloured in yellow. Protein side chains and LPS molecules are shown in CPK sticks representation (cyan – carbon; red – oxygen; blue – nitrogen; brown – phosphorus; sulphur – yellow).
